# Accelerating the sustainable development goals through microbiology: some efforts and opportunities

**DOI:** 10.1099/acmi.0.000112

**Published:** 2020-03-23

**Authors:** Omololu E. Fagunwa, Afolake A. Olanbiwoninu

**Affiliations:** ^1^​ Department of Food and Drugs, Federal Ministry of Health, Abuja, Nigeria; ^2^​ School of Applied Sciences, Biological and Geographical Department, University of Huddersfield, Huddersfield, UK; ^3^​ Department of Biological Sciences, Laboratory of Food and Industrial Microbiology, Ajayi Crowther University, Oyo, Nigeria

**Keywords:** SDGs, sustainability, microbiology, poverty, climate change, Environment

## Abstract

Modernization has thrown humanity and other forms of life on our planet into a ditch of problems. Poverty, climate change, injustice and environmental degradation are a few of the shared global problems. The United Nations Sustainable Development Goals (SDGs) are the blueprint to achieve a better and more sustainable future for all. The SDGs are well structured to address the global challenges we face including poverty, inequalities, hunger, climate change, environmental degradation, peace and justice. Five years into the implementation, the SDGs have been driven mainly by international donors and ‘professional’ international development organizations. The world is left with 10 years to achieve these ambitious goals and targets. Various reviews show that little has been achieved overall, and the SDGs will not be a reality if a new strategy is not in place to bring inclusion. Microbiology, the scientific discipline of microbes, their effects and practical uses has insightful influence on our day-to-day living. We present how microbiology and microbiologists could increase the scorecard and accelerate these global goals. Microbiology has a direct link to achieving SDGs addressing food security, health and wellbeing, clean energy, environmental degradation and climate change. A non-classical growing relationship exists between microbiology and other SDGs such as peace, justice, gender equality, decent work and economic growth. The pledge of ‘Leave No One Behind’ will fast track progress and microbiology is in a better position to make this work.

## Why consider microbiology and SDGs?

Modernization has thrown humanity into the ditch of self-created problems, which they have no ability to solve. Depletion and pollution of our natural resources and inability to maintain human life on Earth due to several health issues, are demeaning the living conditions of several billions of citizens of the world. Numerous solutions have been recommended, which are either not applied or are not suitable for solving the problem at hand. In order to use sustainable development goals (SDGs) as a means of improving human conditions, there is the need to use sustainable approaches, which include the use of micro-organisms, i.e. microbiology.

**Fig. 1.**
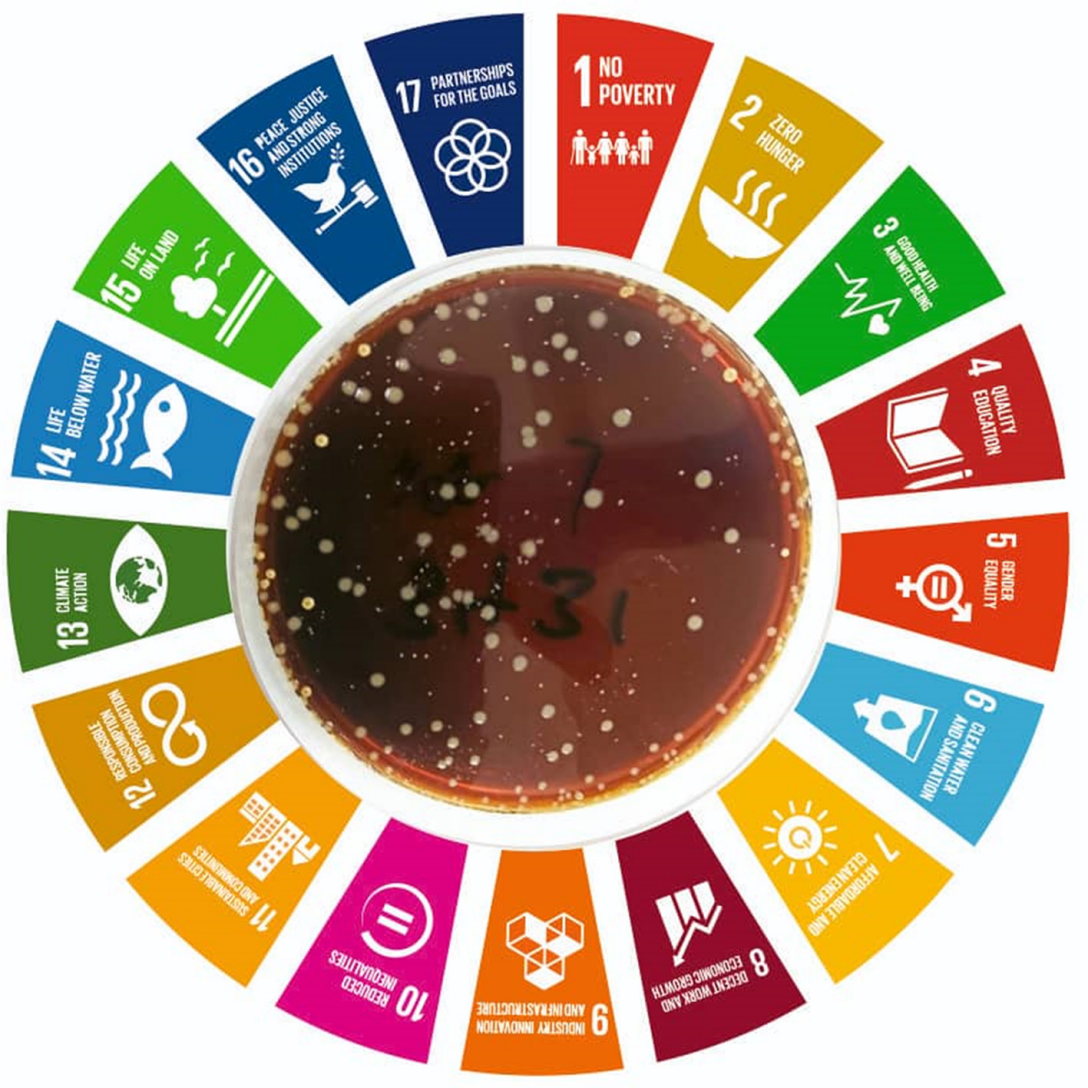
Microbiology and SDGs. Interconnection of microboiology with each of the 17 SDGs.

The SDGs comprise 17 goals and 169 targets aimed at addressing the economic, social and environmental challenges faced by all humans thereby transforming our world and providing a sustainable future for all. Microbiology is the study of microscopic living organisms that have insightful influence on our day-to-day living. This concerns our health, food security, environmental sustainability, climate change, bio-remediation, alternative environmental friendly fuel, improvement of water quality, discovery of new drugs and sustainable food production. All of these are what SDGs are targeting. Therefore, the actualization of all SDGs cannot be achievable without microbiology (Fig. 1) .

## The 17 SDGs

The 17 goals and 169 targets are interconnected with trade-offs and synergies. Synergies, defined by positive correlations between indicator pairs, outweighs trade-offs (negative correlations) for most SDGs and countries. For instance, SDG 1 (no poverty) was found to depict synergies with most goals while SDG 12 (responsible consumption and production) shows trade-offs. In most countries and populations, SDG 3 (good health and wellbeing) has a positive correlation with other SDGs [1]. This body of systematized work using the official SDG indicator data for 227 countries proffer a forward thinking that the synergies can be leveraged on while the trade-offs may need a change in strategies.

## SDG1 – no poverty

Globally, about 600 million people live in extreme poverty. The sustainable goal target is to get 1.7 people/s out of extreme poverty, but the current escape rate is 0.5 people/s. As of October 2019, for every 45 people that escape poverty 15 will fall into a poverty ditch [2]. Impoverished countries in South America and Asia are on track with the SDG target for everyone to escape poverty. However, most countries in Africa are off-track or experiencing a rapid rise in poverty [3]. Poverty has a correlation with the business of microbiology. For instance, a study in a developing country found poverty as an independent risk factor for in-hospital mortality in community-acquired pneumonia (CAP) [4]. The study acknowledged that while numerous factors may affect the prognosis of CAP, poverty stands as an independent factor. A similar study by McLaughlin *et al*. concluded that CAP has an economic burden [5]. Furthermore, the antimicrobial resistance economic cost is driving developing countries into deeper poverty [6, 7]. This economic burden ranged from $21.832 per case to over $3 trillion global loss in gross domestic product (GDP). Another systematic study on poverty and AMR in humans concluded that antimicrobial resistance is interlinked with poverty [8]. The relationship between poverty and infectious disease is problematic since infectious diseases do not respect socio-economic status. Poor people have greater exposure to risk factors of disease and have been economically disadvantaged, which often makes the situation worse (see box 1). Infectious diseases of poverty (IDP), a term used to describe a number of diseases that are prevalent among poorer people include tuberculosis, malaria, HIV/AIDS and the neglected tropical diseases [9]. There is a complex association between malaria and poverty, and the odds of malaria is double in children with the lowest socio-economic position [10]. Microbiologists who are committed (or will commit) to research to stop AMR, discovery of new effective and sustainable antimicrobials including malaria vaccines and other eliminatory interventions are fighting poverty. The field of microbiology is already making efforts in fighting poverty, perhaps it is time that we have more deliberate, conscious efforts in the current and future research and development.

Box 1. **Bitrus’s story: fictionalised story of real issues confronted by millions in extreme poverty**

Akwanga, a solid mineral-rich region that attracts migrant workers from neighbouring states has few functioning government services, civic unrest and experiences environmental degradation due to mineral exploitation. The indigenous people were displaced by mining activities and recently by the Fulani herdsmen whose cattle destroy farms – a livelihood for the locals. Bitrus, picked up mining work since his farmland was destroyed by cattle. Recently, Bitrus began to develop recurrent fever. Believing it is the ‘normal’ malaria, Bitrus bought antimalarial tablets from the ‘chemist’ that serve the miners. Despite taking the medicine, Bitrus fever symptoms persists for days. After 3 days of worse condition, Bitrus sought help at the closest health clinic in a small town, which is about 25 km away. Motorcycles are the only transportation mode to the town and Bitrus had used most of his wages on transport fare, but when he found the clinic, it was closed. The next day, clinic staff gave Bitrus paracetamol, but fever still persisted. The last option for Bitrus was to go back to his family (about 20 km away). There his wife borrowed money to get a private doctor to treat him. The doctor diagnosed Bitrus with HIV, some neurological symptoms, ascariasis, pulmonary tuberculosis and late-stage sleeping sickness (trypanosomiasis). Bitrus's wife tried to raise money to pay for the treatment by selling household assets – a bike and the remnant farmland she uses for subsistence farming to feed the children. Afterwards their daughters were unable to attend school as the situation became increasingly tight for the family.

Akwanga, a solid mineral-rich region that attracts migrant workers from neighbouring states has few functioning government services, civic unrest and experiences environmental degradation due to mineral exploitation. The indigenous people were displaced by mining activities and recently by the Fulani herdsmen whose cattle destroy farms – a livelihood for the locals. Bitrus, picked up mining work since his farmland was destroyed by cattle. Recently, Bitrus began to develop recurrent fever. Believing it is the ‘normal’ malaria, Bitrus bought antimalarial tablets from the ‘chemist’ that serve the miners. Despite taking the medicine, Bitrus fever symptoms persisted for days. After 3 days of worse condition, Bitrus sought help at the closest health clinic in a small town, which is about 25 km away. Motorcycles are the only transportation mode to the town and Bitrus had used most of his wages on transport fare, but when he found the clinic, it was closed. The next day, clinic staff gave Bitrus paracetamol, but fever still persisted. The last option for Bitrus was to go back to his family (about 20 km away). There his wife borrowed money to get a private doctor to treat him. The doctor diagnosed Bitrus with HIV, some neurological symptoms, ascariasis, pulmonary tuberculosis and late-stage sleeping sickness (trypanosomiasis). Bitrus's wife tried to raise money to pay for the treatment by selling the household assets – a bike and the remnant farmland she uses for subsistence farming to feed the children. Afterwards their daughters were unable to attend school as the situation became increasingly tight for the family.

## SDG 2 – zero hunger

Ending hunger and all forms of malnutrition by 2030 is an immense challenge as more than 820 million people do not currently have enough food to eat and the hunger trend is rising fastest [11, 12]. Microbiology may play a key role in ensuring that the over 7 billion people in the world have access to safe and nutritious food. Food is a basic human right, however, the increase in the world's population is exacerbating the current food crisis, however, microbiology is and will continue to reduce this food problem through the improvement of current food production, safety and preservation. At the heart of food security and safety is the production of enough safe food for all. It is forecasted that the world's population will grow to 9.7 billion in 2050 (26 % increase), and sub-Saharan Africa will double by the same year while Europe and North America will experience a 2 % population increase by 2050. World rapid population growth will present challenges for attaining SDGs 1, 2, 3, 4, 5 and 10 [13]. To meet the food demand in 2050, a 100–110 % increase in global food supply is needed and it is plausible that bacteria can help provide answers on how to meet with ‘operation feed the world’ [14]. Certain bacteria can mobilize the needed minerals for plant health [15]. A better understanding of the relationship between soil chemistry, plants' nutritional needs and specific microbe–plant interaction can result in the development of various microbial additives, which can increase crop yield. Furthermore, gut microbiota has recently been known to control satiety [16–18]. Microbial intervention and innovations that specifically control hunger and/or aid fullness will partly solve the double burden of excessive food intake (resulting in obesity) and low food intake (resulting in malnutrition and hunger) [17, 19]. Harrold *et al*. explained that bacteria regulate the release of a satiety-stimulating hormone and modulate host fat storage. It is worth noting that the hunger issue resides largely in African countries and South East Asian countries. It is at alarming index in Central African Republic, Chad, Zambia, Timor-Leste, Sierra Leone, Haiti, Madagascar and Afghanistan (IFPRI, 2016, GHI, 2019) [20, 21]. The solution proffering the response of microbiologists in those countries will also contribute to reducing hunger to nothing. Microbiology can fight for zero hunger by reducing food spoilage and food-borne outbreaks, increasing food production, and providing innovative alternative sources of energy and nutrients over those persistently available [22].

## SDG 3 – good health and wellbeing

For the first time an attempt has been made to quantify 107 diseases and injuries in every region of the world. The global burden of disease study (GBD) reveals trends in diseases. Microbiology is a major driver in achieving SDG3. Four out of the top eleven causes of early death are directly microbiological – lower respiratory infections, diarrheal, HIV/AIDS, tuberculosis [23]. Antimicrobial resistance (AMR) threatens the achievement of SDG3 since infections caused by microbes cannot be effectively prevented or treated when AMR occurs, and this results in prolonging illness, disability and death [24]. AMR also affects other SDGs – SDG 1 (poverty), SDG 2 (hunger), SDG 6 (sanitation), SDG 8 (economic growth), and we need to apply SDG 12 (responsible production and consumption) and SDG 17 (partnership) to tackle AMR. It was argued in the 2015 global sustainable development report (GSDR) brief that goals three and most of it targets will be impossible to achieve without effective antimicrobials [25]. SDG 3 addresses major health priorities, including sexual, reproductive, maternal, newborn, child and adolescent health, environmental, communicable and non-communicable diseases. Studies are emerging that NCDs are influenced by microbes, for instance gut microbiota and non-communicable inflammatory diseases such as allergy, obesity, asthma [26], microbial infections and arrays of NCDs [27]. The European Union sponsored *‘My New Gut’* project, which ended in 2018, provided scientific evidences that gut microbiota influences metabolic health [28]. How microbiology products such as probiotics support good health is attracting the attention of the general population. Bacterial-mediated therapy has been reported to increase specificity and improve the outcome of cancer and tumour therapy [29]. The understanding that microbes underpin health and diseases of communicable and non-communicable origin gives further tasks to microbiologists in addressing SDG 3.

## SDG 4 – quality education

Microbes and their activities have generally positive effects on the functioning (including health and wellbeing) of human beings, the whole of the biological world, and the entire surface of the planet and its atmosphere [30]. What professionals who are interested in microbes know about the microbes and their activities is different from the public view. In a study in Italy, 60 % of children view microbes as harmful and only 25 % admit their positive role [31]. The gap between the reality of what micro-organisms are and the perception by the general population is a lack of quality microbial education. Microbial knowledge is very conserved among microbiologists. Education is the key that unlocks a world of opportunities with individuals benefitting from what they learn. SDG 4 outlines the need for fair and inclusive education and lifelong learning. Inclusive quality education should enshrine microbial education. Microbiology meets with SDG 4 when it passes the message of micro-organisms being just pathogens to co-inhabitants of our environments (gut, land, ocean, animals etc). A path towards microbiology literacy in society includes enshrining key elements of microbiology into basic education, and multi-stakeholder (microbiologist, educators, science communicators) willingness and action for microbiology literacy [32, 30].

## SDG 5 – gender equality

Gender bias is undermining our social fabric and devalues all of us. Aside being a human right issue, it is a big waste of the world’s human potential. Within the SDG 5 targets, microbial science relates most to target 5.3 (eliminate forced female genital mutilation), target 5.6 (universal access to reproductive health). Microbiologists in regions where FGM is still in practice can assist to meet target 5.3 by sensitizing the handlers, parents and girls about the consequences of the practice, especially acquiring urinary tract infection, which may lead to other severe conditions. Moreover, microbiology can contribute to goal 5 as lower respiratory infections is the all-time leading causes of death globally [33, 34]. Global microbiology societies and their leaderships can contribute to target 5.1 (end discrimination against women and girls) and target 5.5 (leadership and decision-making) through increasing involvement of female microbiologists in all businesses including conference – conveners, presenters and speakers. Gender equity can be achieved among speakers at microbiology conferences by making the program committee aware of gender statistics, encouraging females to participate in sessions convening and chairing, and giving direct instruction to try to avoid all-male sessions [35]. Worldwide priorities in women’s health have changed from a narrow focus on maternal and child health to a broader framework of sexual and reproductive health. The former president of the World Bank Group commented that there is a need to promote women’s health for sustainable development [36].

## SDG 6 – clean water and sanitation

Sustainable development goal six is concerned with ensuring clean water and sanitation for all. Globally, 785 million people lack basic drinking water service (improved drinking water source with a round trip of up to 30 mins to collect water), including 144 million who depend on surface water. Globally, 2 billion people use drinking water contaminated with faeces [37]. Environmental microbiology is the best positioned of all aspects of microbiology to support SDG 6 (clean water, sanitation and hygiene – WASH). Microbiology can lend its expertise in sensitization, policy making, national and local action plan and development of scientifically proven and affordable WASH products. Microbiologists especially from the areas most affected with meeting up with WASH targets (e.g. sub-Saharan Africa, central and southern Asia) should actively engage with their communities and have a sense of responsibility on delivering a kind of ‘corporate social responsibility’ outside of their traditional science workspace.

## SDG 7 – affordable clean energy

Energy as a dominant contributor to climate change needs to be reversed. In total, 3 billion people still depend on unsustainable sources – wood, coal, charcoal or animal waste for cooking or heating. Increasing the share of renewable energy in the global mix is one of the targets that appeal to microbiology most. Only 17.5 % of total final energy consumption comes from renewable energy (UN, 2019) [38]. Waste and agricultural substrates such as rice bran [39], rice paddy [40] and microbes have been employed in the production of third-generation bio-fuel such as bio-ethanol, bio-diesel, bio-gases and bio-electricity [41, 42]. Exoelectrogens, micro-organisms with the ability to transfer electrons extracellularly, are being researched in the development of microbial fuel cells (MFCs). MFCs are a bio-electrochemical system that holds potential in the production of sustainable energy. MFC borders around employing microbial catabolic activity directly to generate electricity from degraded organic matter [43, 44]. Because of the low-energy output with MFCs from a single microbe, a cocktail of microbes is used for producing higher-voltage-output [45] Shewanella strains, the best studied electrogens are currently being explored in bio-electricity, including powering a future NASA space mission [46–48].

## SDG 8 and SDG 9 – decent work and economic growth, and industry, innovation and infrastructure

From being the cause of economic losses, to being the foundation for large number of established and developing businesses, microbes have enormous but often overlooked impacts on global economics. ‘Microbial business is a good business’ and ‘where there’s bugs, there’s brass and bronze’ are a few of the catchphrases depicting how microbiology contributes to economic growth. Bio-based economy is projected to grow by at least 50 % by 2030 [49]. Microbial bio-technology has exceptional diversity of applications and opportunities for specialization capable of sustaining economic development, industry, innovation and facilitating workforce growth [49]. All the services and products of microbial bio-technology, from fermentation to the most sophisticated molecular manipulations are creating jobs for people. For instance, in developing countries, petty sellers of fermented products such as ogi, atib, fufu, injera, nuno, bussa, iru, ugba, asami, fura, chikwangue (Africa), pitha, tungrymbai, dosa, dhokia, Appam (India), tempeh, kecap (Indonesia), Taba ng talangka, bagoong, puto, burong (Philipines) and various local beers will be out of income if not for the action of microbes. Fermented food preparation and production has moved from ‘for family’ scale to large-scale operations [50]. Microbial business will be a major driver of enterprise and employment creation in the future but to facilitate attainment of SDG 8 support from policy makers, business and investors will be needed [49].

## SDG 10 – reduce inequalities

SDG 10 focus on reducing inequality within and among countries. This paper will not directly align microbiology with this goal since most of the targets therein are finance related. However, microbiologists and microbiological bodies can make a contribution by reducing income inequalities that exist using the same or similar microbial products from different regions. For instance, bio-drinks such as Yakult is a big brand and generates income in the developed world while similar fermented products in Africa (e.g. nunu) has low income generation. Nunu can be improved to meet international standard. A range of African fermented dairy products are yoghurt-like products harbouring rich and valuable microbial diversity predominately lactic acid bacteria and yeasts [51]. A strong partnership between microbiologists, and bio-investors in developed and those in developing countries could reduce inequalities that can generate income. African fermented products contain several beneficial microbes [50, 51]. Likewise, knowledge inequalities can be reduced with more exchange programmes for students, researchers and other faculties from across nations.

## SDG 11 – sustainable cities and communities

The world’s population is increasing, and such growth needs to be matched to accommodate everyone. Our survival and prosperity will require new, intelligent urban planning that creates safe, affordable, resilient cities and communities. Microbiome of built environment (MoBE) will be making a significant contribution on safety, resilience and survival that were enshrined within this goal. MoBE is a growing field of inquiry that studies the communities of micro-organisms found in man-constructed environments [52, 53]. Microbes are invisible inhabitants that are part of and affected by our environment. Consideration should be given to designing built environment with probiotics to support inhabitant health and wellbeing [54, 55]. Similar studies show that occupants that live in the same building over some time will have similar skin microbiome [56]. In another instance, children who grow up on farms have lower rates of asthma compared to the general, non-farm population [57, 58]. Certain groups of bacteria may protect against development of asthma [59]. Furthermore, the potential opportunities for manipulation of the MoBE for improved mental health was proposed [60]. *Bacillus sphaericus* spores enable self-healing of cracks, hence capable of eliminating traditional damage monitoring and repair, and ultimately reducing maintenance cost [61]. Likewise, bacterial microcapsules create a green concrete wall – a living wall or vertical garden [61]. ‘Bio-informed’ design is getting the attention of architects, and scientists can make the merging field of microbiology (microbiome) of built environment real-time relevant and applicable to address health safety, building resilience and sustainability to include reduction in bio-deterioration of building materials [53].

## SDG 12 – responsible consumption and production

The 9.7 billion projection of the world’s population by 2050 will require the equivalent of three planets to provide the natural resources needed to sustain current lifestyle (UN, 2019). Stewardship is been called in water, energy and food consumption. Microbiology affects the attainment of targets 12.3 (halve global per capital food waste), target 12.4 (responsible management of chemicals and waste), target 12.5 (substantially reduce waste generation). Less than 3 % of the world's water is drinkable (fresh water source) and 1 billion people do not have access to fresh water (UN, 2019). Microbiology will continue to make the limited fresh water safe free of harmful microbes, including the harmful cyanobacterial blooms in freshwater ecosystems [62]. Micro-organisms have a long tradition in the large-scale production of chemical commodities like amino acids, organics acids and bioactive compounds such as antibiotics. Microbial fuel cells have not yet achieved the yields to make them economical but with continued research in this field its potential can be fully released to inspire the next-generation factories of chemical [63]. Nanoparticles have antimicrobial, agriculture, drug delivery, environmental, cosmetics, waste treatment, electronics and construction applications [64, 65]. The production of nanoparticles from chemical and physical methods does not fit into the SDG compared to microbial synthesizes. The former result in high-energy consumption, large waste generation and sometimes toxic while the later does not require solvent, harmful chemicals and starting materials [66]. Kappler and He argued that microbial bio-technology is the future of the sustainable recovery of precious metal from waste streams [67].

## SDG 13 – climate action

Weather patterns are changing, rising sea levels, extreme weather and increasing emission of greenhouse gases are part of the climate change affecting every country in every continent (UN, 2019). Climate change is disrupting of national economies and affecting lives, people, communities today and in the future. The International Panel on Climate Change's (IPCC) recent report shows that limiting warming to 1.5 °C rather than the 2 °C or higher is beneficial and possible within scientific laws in chemistry and physics but will require unprecedented transitions in all aspects of society [68]. Microbes, the ‘unseen majority’ play a role in climate change notwithstanding – microbes in climate-change response. The consensus statement published by 34 microbiologists across nine countries warned that micro-organisms play a central role and of global importance in the biology of climate change. The document also noted that the impact of climate change will depend heavily on microbial response [69]. Microbiology works hand in hand with SDG targets 13.1 (strengthen resilience and adaptive capacity to climate-related hazards and natural disasters in all countries), target 13.2 (integrate change measures into national policies, strategies and planning), and target 13.3 (build knowledge and capacity to meet climate change). Adaptivity and resilience of humans and other life forms on earth to anthropogenic climate change will be achieved by incorporating microbial knowledge since micro-organisms support the existence of all higher life forms. Microbiology is essential for achieving an environmentally sustainable future. Cavicchioli *et al.* call on microbiologists to raise microbial-climate-change awareness and engage with microbial research that can be increasingly integrated into the frameworks for addressing climate change and accomplishing the UN SDGs [69].

## SDG 14 – life below water

Ocean and sea cover 70 % of our planet and we rely on them for energy, food and water. However, these precious resources have been damaged by humans (UN, 2019). Microbiology as a discipline that has lots of interaction with seas and oceans can reduce marine pollution (target 14.1), protect and restore the ecosystem (target 14.2), reduce ocean acidification (target 14.3), increase the economic benefits from sustainable use of marine resources – aquaculture (target 14.7) and increase scientific knowledge for ocean health (target 14.A). The ocean absorbed about 30 % of the anthropogenic CO_2_, and this result to ocean acidification. Ocean acidification will impact on the growth, development, calcification, survival and abundance of a range of species, from algae to fish [68]. Acidification is adding pressure on global food security as sustainable fisheries and aquaculture will be difficult to attain under this acidic condition [68]. Coral-reef degradation could be mitigated by manipulation of coral-associated prokaryotes. For instance, specific taxa that can degrade oil can by inoculated, conferring health to corals and better water quality [70]. Synthetic plastics continue to be a threat to oceans, and different ways are being considered to reduce their impact. A way is the use of micro-organisms that are capable of degrading plastic and micro-organisms isolated from cold marine is a major consideration to solving this problem because they have higher bio-degradation rate [71]. Examples of microbes with plastic degrading capability includes *Shewenella*, *Moritella *, *Phormidium* sp., *Arthrobacter *, *Pseudomonas *, *Vibrio alginolyticus *, Vibrio* parahemolyticus*, *Clonostachys rosea*, *Rhodococcus* sp., *Aspergillus versicolor*, *Alcanivorax* sp., *Tenacibaculum* sp., *Bacteroides *, *Rhodobacter* [71, 72]. A bio-degradable plastic must pass through bio-deterioration (stage 1), bio-fragmentation (stage 2), and assimilation and mineralization (stage 3). In the absence of relevant microbes, stages 1 and 3 cannot occur [72, 73]. Advances in microbial bio-technology are creating possibilities for polymer bio-degradation [74, 75].

## SDG 15 – life on land

Forest loss is slowing down, and more financial assistance is flowing towards bio-diversity, however bio-diversity is still occurring at an alarming rate. More so, about 74 % of the poor are directly affected by land degradation, and 23 hectares is lost every minute due to drought and desertification (UN, 2019). On bio-diversity, the United Nations recognized that micro-organisms and invertebrates are key to ecosystem services, but their contributions are still poorly understood and rarely acknowledged (UN, 2019). The world’s forests are built by symbiotic relationship between trees and micro-organisms [76]. The kinds of dominant root-associated microbial symbionts (fungi and bacteria) in a forest will determine access to nutrients, sequester carbon, and resilience in the face of climate change. Steidinger *et al.*'s study predicted that if carbon emission continues, there will be a 10 % reduction in the bio-mass of tree species associated with fungi, especially in the cooler regions of the world. Another previous study by Zifcakova *et al.* shows microbial activity in coniferous forest soil reflects changes in ecosystem properties between warm and cold seasons [77]. Fungi are important microbes in forest inhabiting and connecting habitats, and involved in other complex ecosystem processes [78, 79]. Could it be said then that fungi fuse the forests? Brownfields that results from soil contaminated with petroleum hydrocarbons can be reversed to make the site safe and sustainable use through integrative bio-remediation approaches that uses microbes. Brownfields contains co-contaminants that pose risks to human and environmental health [80]. How microbes support life on land requires more research to fulfil SDG 15.

## SDG 16 – peace, justice and strong institutions

SDG 16 aim to promote peaceful and inclusive societies where justice prevails, and institutions are strong and effective (UN, 2019). Microbes do not have a direct link in fulfilling this goal since it largely entails laws and governance. However, microbiology could indirectly contribute to justice, peace and security in society through microbial forensic and counter-bio-terrorism. Neuromicrobiology could contribute more to the justice system. Certain microbial infection damages the nervous system to an extent that results in abnormal and inappropriate behaviours. This is in the case of a truck man who was dismissed from his job while there was an underlying factor for his inappropriate behaviours – he was infected with *Bartonella henselae* [81]. *B. henselae* causes cat-scratch disease, which results in neurological complications [82]. The B. *henselae* infection in the truck driver made him have memory loss and after treatment the bacteria DNA was not detected [81]. The truck man may have had a cat bite, but justice prevailed for him because of microbial forensics. Similarly, the parasite *Toxoplasma gondii* may arouse sexual masochism and submission [83, 84]. Microbes can change toxicology results, point to causes of death and match suspects to the scene of a crime [85]. Microbes can keep time for forensic investigators. This microbial clock allows for the identification of microbes at different stages of a body decomposition [85]. Further investigation and research in different seasons and environments needs to be done to have this forensic tool deployed in crime scenes and courtrooms. Bio-terrorism and bio-crimes though rarely happening could be checked and countered with advances in microbial forensics [86–88]. Chauhan *et al.,* Schutzer *et al.* [87, 89] and Beans document further examples of association between microbes and peace and justice. Systems and methods that detect, track or even prevent biological attacks will improve security.

## SDG 17 – partnership for the goals

**Fig. 2.**
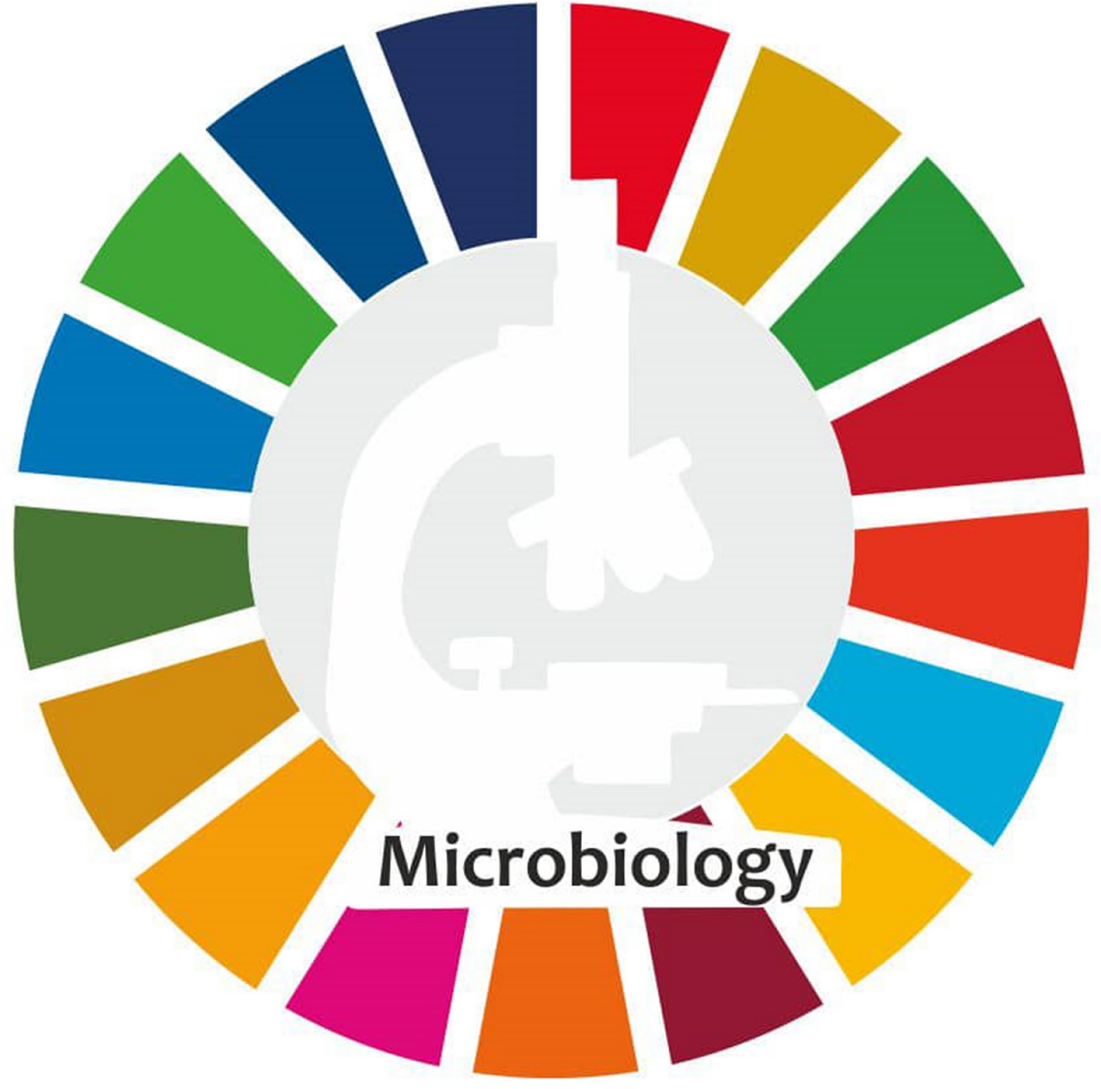
Centrality of the study of microbes to attaining the global goals.

The global goals can be met if we all work together. Microbiology can have a strong partnership for the goals through knowledge sharing and cooperation for access to science, technology and innovation (targets 17.6), promotion of sustainable technologies to developing countries (target 17.7), strengthening of science, technology and innovation capacity for the least developed countries (target 17.8) and encouragement of effective partnerships (target 17.17). Microbial technologies can be a means for microbiology to establish or revitalize global partnership for sustainable development. More microbiology partnerships can be promoted within and across countries (Fig. 2).

## Gaps, challenges and recommendation

### Gaps and challenges

Despite the vast promise of microbes to contribute to achieving the ambitious sustainable development goals, there is still a lot to consider as gaps and challenges. There is a gap within the scientific community about what SDGs are about. This gap is broader among the developed nations, perhaps because of low or no public education, information and sensitization within those countries. Many of these countries may not be most affected with a development goal, e.g. poverty, extreme hunger, quality education, industry, innovation and infrastructure. In the UK for instance, SDGs are synonymous to the climate-change campaign. However, SDGs are beyond climate change, despite the latter having a weight on the phenomena. Moreover, the InterAcademy Partnership survey among expert members of merit-based academies, the global network of science, engineering and medical academies reported limited awareness of SDGs among members [90]. There is one Earth which is so diverse not in terms of people and language, culture or tribes but it is also microbiologically diverse. There is still more to do on research and development. The inequality in research capacities and development among nations and regions will slow the contributions of microbial science to SDGs. Despite the widespread growth in researchers, little change in the global balance. EU, China, Japan, USA and Russia account for 72 % of global researchers. As at 2014, the entire continent of Africa produces only 2.6 % of global scientific publication, whereas Asia (39.5%), Europe (39.3%) and Americas (32.8%) get most of the figure. Scientific imbalance also exists among regions, for instance, China alone account for over half of the entire science in Asia, whereas the entire Central Asia sub-region only accounts for less than 0.3 % [91]. Aside from the inequalities in research and development capacities and output, some microbes are laying in abundance somewhere to be isolated, characterized, elucidated in terms of benefits and harmful potentials. Hence, a complete microbial transcriptome will help. A complete database of microbes present in our environment, in and out of seasons can help us elucidate the potential and implication of these microbes in various ecological, bio-technological and industrial processes.

### Recommendations

The starting point to effectively gathered partnership, inclusiveness and ‘no one left behind’ among microbiologists and associates is to saturate the scientific community with education and information about SDGs.

Scientific conferences can be reprogrammed around promotion of SDGs. That will afford presenters and speakers to align their work with the global commitment. Recognitions and awards can be given to researchers whose works have the most impact and are ground breaking on achieving any sustainable goals or targets to reflect the SDGs in programmes and initiatives. Microbiology societies within each country and regional and international societies such as Microbiology Society, Federation of European Microbiological Society, American Society of Microbiology, International Union of Microbiological Societies may lead the way in considering and implementing these recommendations.

Furthermore, a dedicated SDG fund may be allocated within the treasuries of microbiological societies, biological funding bodies, and university departments to foster microbiology-SDGs outreach, SDG-aligned research, or any of such activities. Likewise, the research and development inequalities should be reduced if not erased among microbiologists within and across countries. This will rest mainly on the shoulder of the national government, then on funding bodies, universities/institutes, industry partners, regional/international societies. An imminent way forward on this is through science diplomacy.

At a high level, microbiology can help support the SDGs either through national societies or various merit-based science academies where microbiologists are embedded by

- Participating in the annual UN Science Technology and Innovation multi-stakeholder forum.
- Timely response to calls for input to the Global Sustainable Development Report (GSDR).
- Contributing to the development of STI roadmaps/action plans at national level.
- Providing relevant expertise to the science-based work of different UN structures and national departments and agencies.

It will be a worthwhile project for microbiological societies to have a survey among members on the SDGs. The actual and aspirational role of microbiologists and associates in supporting the SDG framework can be learned. Survey questions can be tailored to understand current knowledge and awareness, where microbiology–SDG engagement is and should be, and where capacities should be developed.

### Conclusion

The SDGs are a group of 17 overall goals with 169 targets aimed at improving life on Earth, specifically to end poverty, protect the planet, and ensure prosperity for all and to solve most serious global economic, societal and environmental issues. The SDGs are highly ambitious, admirable, and more than just a little hopeful, but are they in any way achievable by their 2030 deadline? The microbiological community is well-placed to take a lead in tackling many of the SDG targets: beating infectious disease, provision of clean water, food security, maintenance of bio-diversity in the oceans and on land, and uptake of green energy such as bio-fuels. With some lateral thinking, microbiology is relevant to all the SDGs. The multidisciplinary nature of microbiological research will help in facilitating an integrated approach to answering questions and solving problems raised by the SDGs.
