# Corrigendum: Accelerating the sustainable development goals through microbiology: some efforts and opportunities

**DOI:** 10.1099/acmi.0.000174

**Published:** 2020-12-21

**Authors:** Omololu E. Fagunwa, Afolake A. Olanbiwoninu

**Affiliations:** ^1^​ Department of Food and Drugs, Federal Ministry of Health, Abuja, Nigeria; ^2^​ School of Applied Sciences, Biological and Geographical Department, University of Huddersfield, Huddersfield, UK; ^3^​ Department of Biological Sciences, Laboratory of Food and Industrial Microbiology, Ajayi Crowther University, Oyo, Nigeria

The figures used in this manuscript contravened the copyright legislations of The United Nations (UN) regarding permissions to reuse images representing their Sustainable Development Goals (SDGs).

Fig. 1 incorrectly used the UN’s SDGs imagery as the background to their figure without citation or permission. The figure should have not included the imagery and should have been displayed as follows:


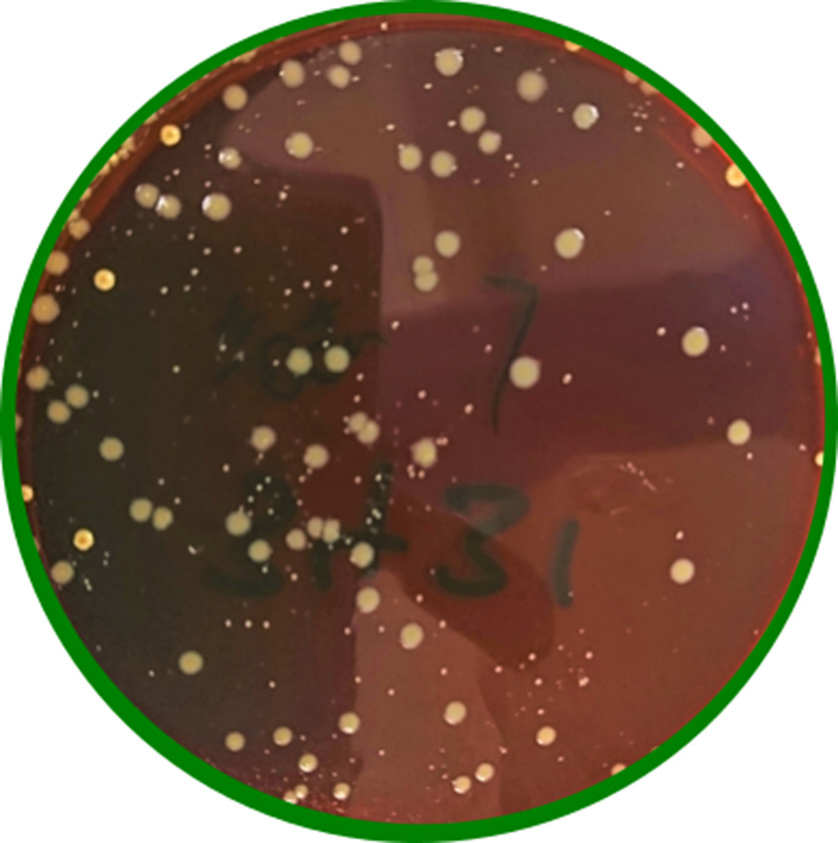


Fig. 2 contained an image displaying the UN’s SDGs which non-UN affiliations are not permitted to re-use. The figure should have displayed the following reusable image:


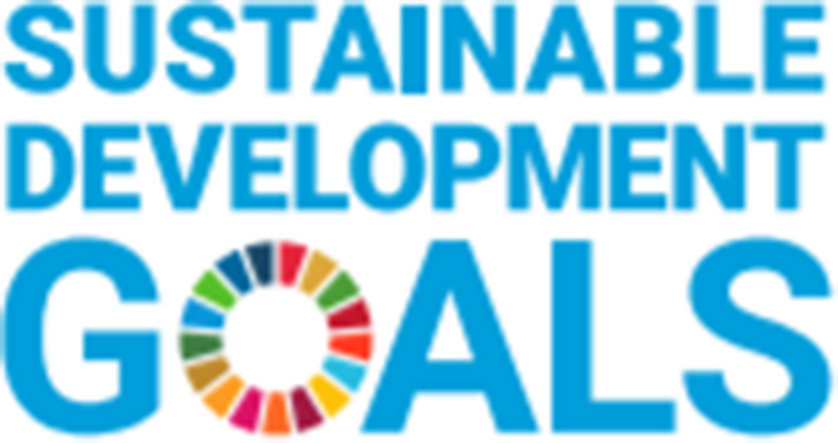


The authors apologise for any inconvenience.

